# Antimicrobial Activities of Ellagitannins against *Clostridiales perfringens*, *Escherichia coli*, *Lactobacillus plantarum* and *Staphylococcus aureus*

**DOI:** 10.3390/molecules25163714

**Published:** 2020-08-14

**Authors:** Elina Puljula, Gemma Walton, Martin J. Woodward, Maarit Karonen

**Affiliations:** 1 Natural Chemistry Research Group, Department of Chemistry, University of Turku, FI-20014 Turku, Finland; elina.puljula@gmail.com; 2 Department of Food and Nutritional Studies, The University of Reading, Reading RG6 6AH, UK; g.e.walton@reading.ac.uk (G.W.); m.j.woodward@reading.ac.uk or; 3 Folium Science, Unit-DX, Bristol BS2 OXJ, UK

**Keywords:** antibacterial, growth inhibition, hydrolysable tannins, polyphenols

## Abstract

In this study, we tested the growth inhibition effect of 22 individual ellagitannins and of pentagalloylglucose on four bacterial species, i.e., *Clostridiales perfringens, Escherichia coli, Lactobacillus plantarum* and *Staphylococcus aureus*. All tested compounds showed antimicrobial effects against *S. aureus*, and almost all against *E. coli* and *C. perfringens*. For *L. plantarum*, no or very weak growth inhibition was detected. The level of inhibition was the greatest for *S. aureus* and the weakest for *C. perfringens*. For *S. aureus*, the molecular size or flexibility of ellagitannins did not show a clear relationship with their antimicrobial activity, even though rugosins E and D and pentagalloylglucose with four or five free galloyl groups had a stronger growth inhibition effect than the other ellagitannins with glucopyranose cores but with less free galloyl groups. Additionally, our results with *S. aureus* showed that the oligomeric linkage of ellagitannin might have an effect on its antimicrobial activity. For *E. coli*, the molecular size, but not the molecular flexibility, of ellagitannins seemed to be an important factor. For *C. perfringens*, both the molecular size and the flexibility of ellagitannin were important factors. In previous studies, corilagin was used as a model for ellagitannins, but our results showed that other ellagitannins are much more efficacious; therefore, the antimicrobial effects of ellagitannins could be more significant than previously thought.

## 1. Introduction

Dietary tannins can affect animal nutrition and health in several ways, for example, by improving the uptake of amino acids from feed proteins, by increasing anthelmintic effects against parasitic intestinal nematodes, and by lowering gaseous ammonia and methane emissions [1,2,3,4,5]. Tannins have also been shown to possess antimicrobial effects against several pathogens [6,7,8] and have been studied as an alternative for antibiotics or synthetic anthelmintics [9,10]. 

Tannins, i.e., condensed tannins (syn. proanthocyanidins), hydrolysable tannins including simple galloyl derivatives, gallotannins, ellagitannins (ETs), and phlorotannins are a unique group of plant specialized metabolites with diverse structures [11]. They have the well-known ability to form complexes with proteins and precipitate them [12]. In general, their molecular size, conformational mobility and flexibility in addition to the affinity of the polyphenol for water are important factors influencing the capacity of tannins to interact with and bind to proteins. Different tannins can have very different bioactivities depending on the exact tannin structure and, for example, ETs can establish simultaneously strong and weak interactions with proteins [13]. The anthelmintic activity of ETs against *Haemonchus contortus* and *Trichostrongylus colubriformis* third-stage infective larvae is increased as the molecular size of tannin increases [10], whereas the anthelmintic activity of hydrolysable tannins against egg hatching and motility of L1- and L2-stage *H. contortus* larvae indicate a clear relationship between the structure of hydrolysable tannins and the activity observed [14]. No clear relationship was observed between activity and molecular size, but the optimal structure was found for pentagalloylglucose. The anthelmintic properties of hydrolysable tannins vary depending, for example, on the structure of tannins, the parasite species and their growth stages [10,14,15,16]. Similarly, the antimicrobial effects of tannins seem to be species-specific and linked to tannin structure, not only to their ability to precipitate proteins [9].

Most of tannin-related antimicrobial studies have been conducted on plant extracts instead of pure compounds, making it difficult or impossible to compare the antimicrobial activity of individual compounds or to assess structure–activity relationships. Additionally, several mechanisms of action have been proposed through which tannins can induce their antimicrobial effects [9]. So far, only few studies have tested the antimicrobial activity of individual ETs [17,18,19,20]. Kolodziej et al. (1999) [18] tested the antimicrobial activities of tannin and related compounds and noticed that the ETs corilagin and phyllanthusiin C were weak inhibitors of *Escherichia coli*, *Klebsiella pneumoniae*, *Bacillus subtilis*, *Pseudomonas aeruginosa*, and *Proteus mirabilis* (with minimum inhibitory concentrations (MICs) of 1000 or 2000 µg/mL). However, corilagin showed moderate activity against *Staphylococcus aureus* (MIC 250 µg/mL). Corilagin inhibits the growth of *E. coli* by disrupting its membrane permeability and that of *S. aureus* by inhibiting protein expression [21]. In addition, Taguri et al. (2006) found that castalagin had a comparatively strong antibacterial activity against several bacteria species, and punicalagin had a moderate activity in comparison to other polyphenols [19]. Funatogawa et al. (2004) tested altogether 21 individual ETs together with pentagalloylglucose against *Helicobacter pylori* and *E. coli* (JC-2) and found that all tannins tested showed promising antibacterial activity against *H. pylori* and that monomeric ETs revealed stronger antibacterial activity than ET oligomers [20]. The tannins examined did not reveal any antibacterial activity against *E. coli* at the tested concentrations (maximum tannin concentration 100 µg/mL).

**Figure 1:**
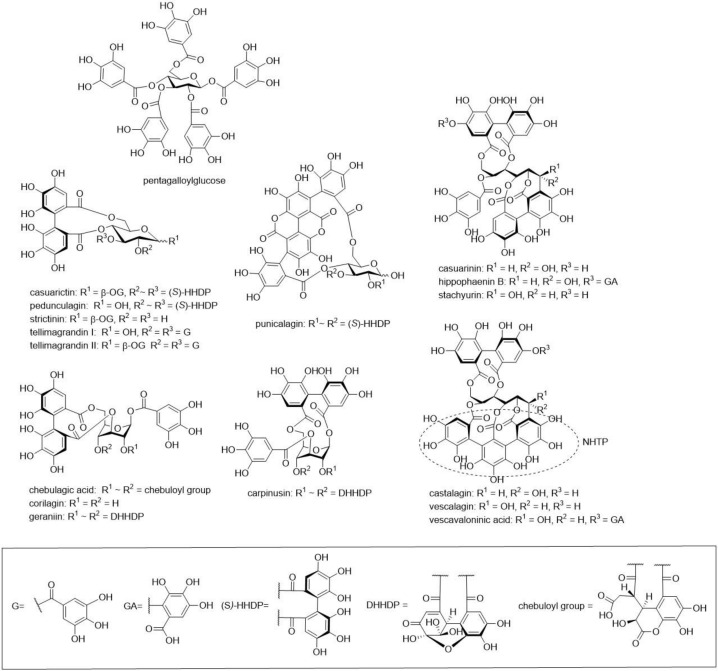
Structures of monomeric ellagitannins and of pentagalloylglucose utilised in this study.

**Figure 2:**
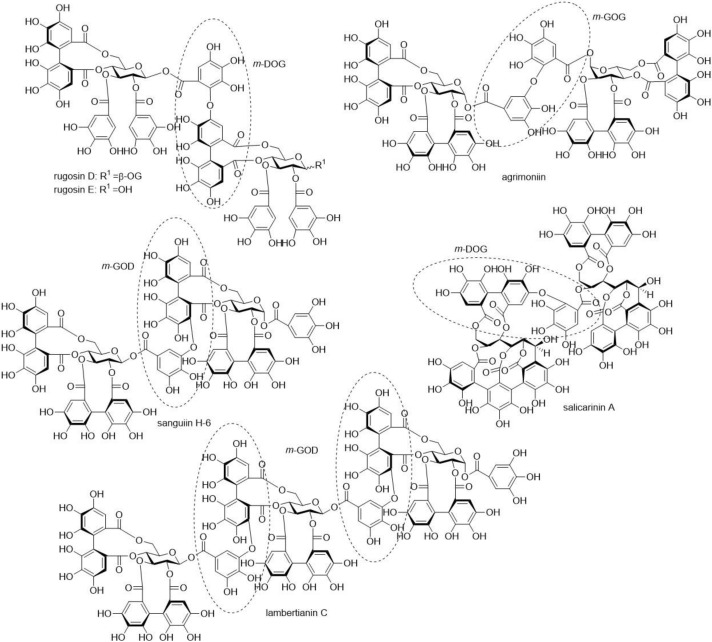
Structures of oligomeric ellagitannins utilised in this study.

Our study analysed the antimicrobial activity of individual ETs against four different bacteria species: *E. coli*, *S. aureus*, *C. perfringens* and *L. plantarum*. *E. coli* is a Gram-negative bacterium, whereas *S. aureus*, *C. perfringens* and *L. plantarum* are Gram-positive. All four species were used previously in tannin-related antibacterial studies. Twenty-two ETs were selected, so that they comprised monomeric hexahydroxydiphenoyl (HHDP) esters, monomeric dehydroHHDP (DHHDP) esters and acyclic C-glucosidic ET monomers, including also nonahydroxytriphenoyl (NHTP) esters and ET dimers and trimers with varying types of bonds between the monomers and with acyclic or cyclic glucose cores (see structural examples in Figure 1 and Figure 2). The use of pure compounds was a necessity in order to reveal structure–activity relationships. We wanted to answer the following questions: (i) What are the antimicrobial effects of ETs? (ii) What are the structural features of ETs that affect their antimicrobial activity? (iii) Do ETs have unwanted antimicrobial effects against probiotics?

## 2. Results and Discussion

### 2.1. Inhibitory Effects of Ellagitannins against S. aureus

**Figure 3:**
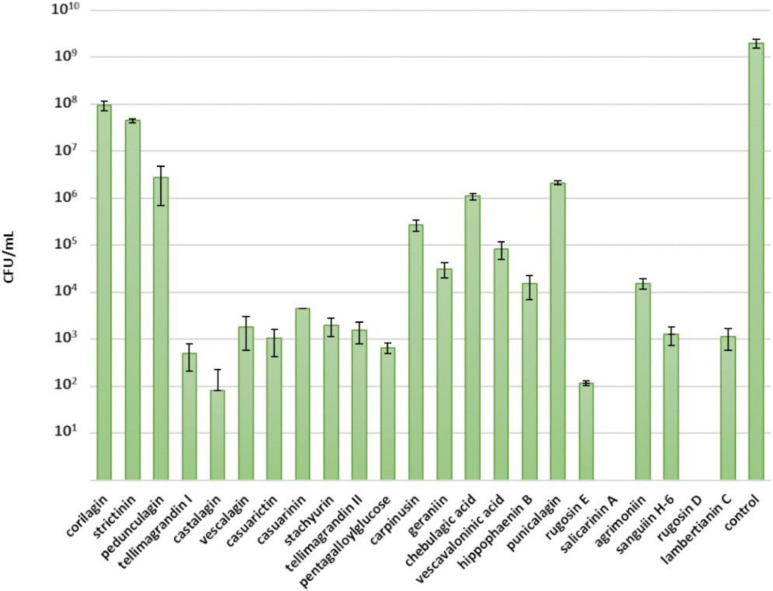
Growth of *Staphylococcus aureus* following a 24 h treatment with 0.5 mM ellagitannin and pentagalloylglucose solutions. Growth is expressed as number of colony-forming units (CFU) per mL (CFU/mL, mean values and standard deviations, *n* = 3). All 23 tested tannins are in the order of increasing molecular weight (CFU/mL in base 10 logarithmic scale); *p* < 0.05 for all compounds.

The tested compounds showed the greatest inhibition against *S. aureus* (Figure 3). Salicarinin A and rugosin D inhibited the growth of *S. aureus* completely at a 0.5 mM concentration. Casuarictin, tellimagrandins I and II, pentagalloylglucose, stachyurin, casuarinin, vescalagin, castalagin, rugosin E, sanguiin H-6 and lambertianin C showed efficient inhibition as well, as a tannin concentration of 0.5 mM slowed growth and total bacterial yield (Figure 3). Previously, Taguri et al. (2006) reported castalagin to be a stronger inhibitor (MIC 267 µg/mL) than other polyphenols against *S. aureus*, while punicalagin showed a moderate antibacterial activity (MIC 600 µg/mL) [19].

Rugosin D, which consists of two tellimagrandin II monomers connected by an *m*-DOG-linkage, was more efficient than the corresponding monomer and, similarly, rugosin E, which consists of tellimagrandin I and tellimagrandin II monomers, was more efficient than the corresponding monomers at restricting the growth of *S. aureus*. Correspondingly, salicarinin A, which is a dimer of vescalagin and stachyurin connected by an *m*-DOG-linkage, was more efficient than its monomers at restricting *S. aureus* growth. However, no trend was clearly discernible when comparing the antimicrobial activity with the molecular weight of the ETs.

We also looked at the effects of other structural features of ETs in detail. The monomeric HHDP esters tellimagrandin I and II and casuarictin had similar inhibitory effects against *S. aureus* as the C-glucosidic ET monomer vescalagin (Figure 3). Correspondingly, dimeric ETs with a glucopyranose cores, namely, rugosins E and D, were similar inhibitors as the C-glycosidic dimer salicarinin A. This suggests that the configuration of the central glucose core of ET does not affect its antimicrobial activity, whereas the type of the oligomeric linkage may play a role in the antimicrobial activity. The ET dimers with an *m*-DOG linkage, namely, salicarinin A and rugosins E and D, exhibited stronger inhibition than the ET oligomers with *m*-GOD linkages, namely, sanguiin H-6 and lambertianin C. The dimeric ET agrimoniin with an *m*-GOG-linkage was clearly a less effective inhibitor than the other dimeric ETs with *m*-DOG and *m*-GOD linkages.

The role of free galloyl groups in the ET structure was not so straightforward. The tannins with glucopyranose cores having four or five free galloyl groups, i.e., rugosins E and D and pentagalloylglucose, were more efficient inhibitors of *S. aureus* than the other cyclic ETs with less free galloyl groups. Tellimagrandin II, with an additional galloyl group, was a weaker inhibitor in comparison to tellimagrandin I, whereas rugosin D, with an additional galloyl group, was a stronger inhibitor in comparison to rugosin E. Interestingly, the acyclic ETs having no free galloyl groups, such as castalagin and salicarinin A, also showed efficient inhibition. Acyclic ETs have NHTP groups in their structures instead of free galloyl groups (Figure 1) and, therefore, are rather rigid. Previous studies have shown that molecular flexibility is an important factor for the bioactivity of ETs [10,13,14]. For example, our anthelmintic results clearly indicated that the intramolecular oxidative coupling that results in the formation of HHDP and NHTP groups decreased the inhibitory activity of hydrolysable tannins against egg hatching of *H. contortus* [14]. Based on our current observations, it seems that molecular flexibility does not have a significant role in the antimicrobial activity of ETs against *S. aureus*. Castalagin, with an NHTP group, was a stronger inhibitor than casuarinin, which has galloyl and HHDP groups, and, similarly, vescalagin, with an NHTP group, was a stronger inhibitor than stachyurin, with galloyl and HHDP groups.

The orientation of the hydroxyl group at the C-1 position in C-glycosidic ETs did not seem to have an effect on the antimicrobial activity against *S. aureus*. Castalagin, with an α-oriented hydroxyl group, was a stronger inhibitor than vescalagin, with a β-oriented hydroxyl group, whereas casuarinin, with an α-oriented hydroxyl group, was a weaker inhibitor than stachyurin, with a β-oriented hydroxyl group.

Monomeric DHHDP esters, i.e., carpinusin and geraniin, showed moderate inhibition of the growth of *S. aureus*: carpinusin was more efficient than geraniin. In general, monomeric ETs with the ^4^C_1_ conformation of central glucose core, namely, corilagin, geraniin, chebulagic acid and carpinusin, showed moderate inhibition. The weakest growth inhibitors of *S. aureus* were strictinin and corilagin. They were the only 2 of the tested 23 compounds that have two free OH-groups in their central glucose unit. Strictinin previously showed weaker anthelmintic activity in relation to other ellagitannins against *T. colubriformis* [10].

### 2.2. Inhibitory Effects of Ellagitannins against E. coli

**Figure 4:**
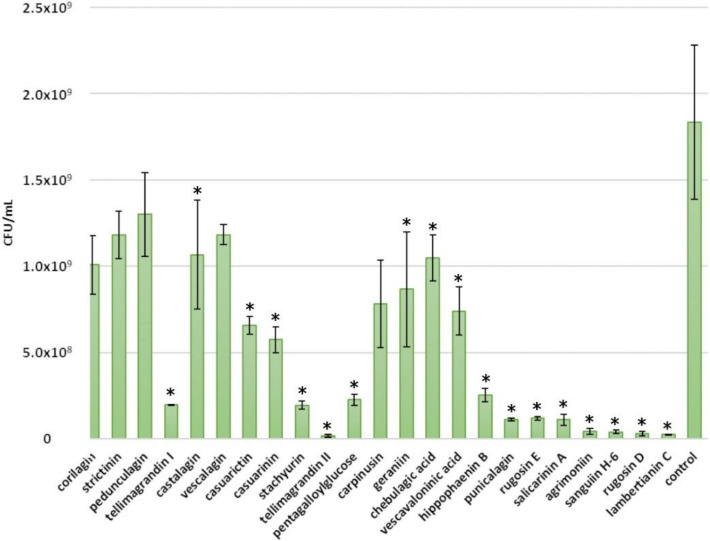
Growth of *Escherichia coli* after a 24 h treatment with 0.5 mM ellagitannin and pentagalloylglucose solutions. Growth is expressed as CFU/mL (mean values and standard deviations, *n* = 3). All 23 tested tannins are in the order of increasing molecular weight; * *p* < 0.05.

Some growth inhibition was also observed in *E. coli*. Almost all tested ETs as well as pentagalloylglucose showed inhibition against *E. coli* (Figure 4). The most efficient compounds were tellimagrandin II, rugosin D, agrimoniin, sanguiin H-6 and lambertianin C. They were able to reduce the growth from 10^9^ CFU/mL to 10^7^ CFU/mL with a 0.5 mM tannin concentration. Previously, it was reported that punicalagin is a weaker inhibitor of *E. coli* (MIC 2133 µg/mL) than castalagin (MIC 533 µg/mL) [19]. Our results did not support this observation, as punicalagin appeared to be a greater inhibitor than castalagin. In the same study, the effects of punicalagin and castalagin were found to be stronger on *S. aureus* than on *E. coli*, similarly to our study. Another study tested pentagalloylglucose, strictinin, pedunculagin, tellimagrandin I, tellimagrandin II, casuarictin, corilagin, geraniin, casuriniin, agrimoniin and rugosin D against *E. coli* (JC-2) and found them not to be active [20].

**Figure 5:**
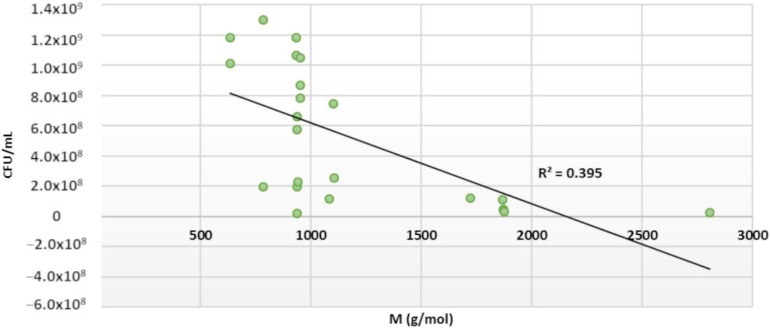
The effect of the molecular size of ellagitannin on its antimicrobial strength against *E. coli* (24 h treatment with 0.5 mM tannin solution). A linear regression model was used.

There seems to be a medium correlation between the molecular size of ETs and the intensity of their antimicrobial activity against *E. coli* (Figure 5). Oligomeric ETs (molecular weight >1700 g/mol) inhibited over 90% of the *E. coli* growth, and their average growth inhibition was 97%. The average growth inhibition for ET monomers was 63%, and the sole monomers with over 90% growth inhibition were punicalagin and tellimagrandin II. Sanguiin H-6, which consists of two casuarictin monomers, and lambertianin C, which consists of two casuarictin and one potentillin monomers, were both more effective than casuarictin. Correspondingly, salicarinin A, which is a dimer of vescalagin and stachyurin, was more efficient than its monomers. However, tellimagrandin II seems to be an exception, as it was more efficient than rugosin D (which consists of two tellimagrandin II monomers) and rugosin E (which consists of tellimagrandin I and II monomers).

The number of free galloyl groups may be an important factor for the antimicrobial effects of ETs against *E. coli*. Additional free galloyl groups enhanced the inhibitory effects of ET: tellimagrandin II and rugosin D were more efficient than tellimagrandin I and rugosin E, respectively. However, the activity of pentagalloylglucose was lower than that of tellimagrandins I and II, indicating the importance of HHDP in the structure.

The effects of other structural ET features on the growth inhibition of *E. coli* were not so clear. The conformation of the central glucose core of ETs did not seem to affect the antimicrobial activity; for example, strictinin with a ^4^C_1_ glucopyranose core, chebulagic acid with a ^1^C_4_ glucopyranose core and castalagin with acyclic glucose had similar activities. The orientation of the hydroxyl group at the C-1 position of ETs did not seem to have an effect on the antimicrobial activity against *E. coli*. The α-anomer castalagin was more efficient than the corresponding β-anomer vescalagin, whereas the α-anomer casuarinin was less efficient than the corresponding β-anomer stachyurin. The formation of the HHDP group by oxidative coupling from two galloyl groups did not explain these differences. Casuarictin was more efficient than strictinin, whereas pedunculagin was less efficient than tellimagrandin I. Molecular flexibility did not seem to be important: pentagalloylglucose, with five free galloyl groups, was less efficient than tellimagrandin II, with one HHDP and three galloyl groups, and the acyclic ETs, with rigid NHTP groups, did not differ from cyclic ETs. The oligomeric linkages did not make a difference either, as all ET oligomers were effective against *E. coli*.

### 2.3. Inhibitory Effects of Ellagitannins against C. perfringens

**Figure 6:**
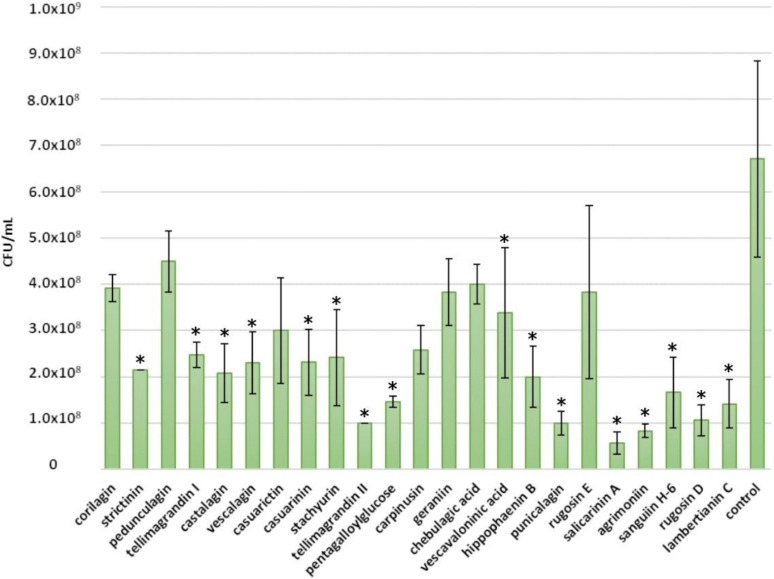
Growth of *Clostridiales perfringens* after a 24 h treatment with 0.5 mM ellagitannin and pentagalloylglucose solutions. Growth is expressed as CFU/mL (mean values and standard deviations, *n* = 3). All tested tannins are in the order of increasing molecular weight; * *p* < 0.05.

Weak inhibition was observed for *C. perfringens*. However, almost all ETs tested showed at least some inhibitory effects. The best inhibitors, salicarinin A and agrimoniin, were able to lower *C. perfringens* growth only to 10^7^ CFU/mL from 10^8^ CFU/mL (Figure 6).

**Figure 7:**
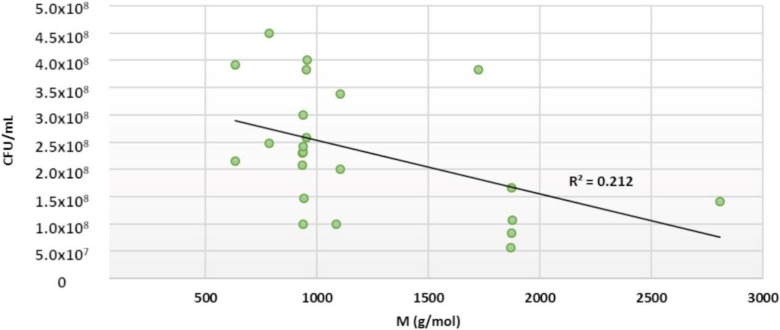
The effect of ellagitannin molecular size on its antimicrobial strength against *C. perfringens* (24 h treatment with 0.5 mM tannin solution). A linear regression model was used.

The molecular size of the ET seemed to be the main explanatory factor for the antimicrobial effects of ETs against *C. perfringens* (a small correlation between the molecular size of ETs and their antimicrobial strength is shown in Figure 7), similarly to *E. coli*. The average growth inhibition for oligomeric ETs was 77%, while for monomeric ETs it was 61%. As with *E. coli*, punicalagin and tellimagrandin II were the most effective monomers (growth inhibition 85%). Compared with other oligomeric ETs, rugosin E showed significantly lower growth inhibition, corresponding only to 43%. Again, it was noticed that salicarinin A, which is a dimer of vescalagin and stachyurin connected by an *m*-DOG-linkage, was more efficient than its monomers. Additionally, sanguiin H-6 (casuarictin dimer) and lambertianin C (casuarictin trimer) were both more effective than casuarictin. However, the same was not observed for dimeric rugosins E and D, as rugosin D was as effective as its monomer, tellimagrandin II, and rugosin E was a weaker inhibitor than its monomers.

The number of free galloyl groups may be an important factor for the antimicrobial effects of ETs against *C. perfringens*. The additional free galloyl group enhanced the inhibitory effects of the ET: tellimagrandin II and rugosin D were more efficient than tellimagrandin I and rugosin E, respectively. The other structural features of ETs did not show any overall clear effect. It was reported that, according to the MIC values, the antibacterial activities of punicalagin and castalagin are stronger against *C. perfringens* than against *S. aureus* [19]. Our results indicate the opposite; however, a comparison between our and this study is difficult, as different methods and strains were used.

### 2.4. Inhibitory Effects of Ellagitannins against L. plantarum

**Figure 8:**
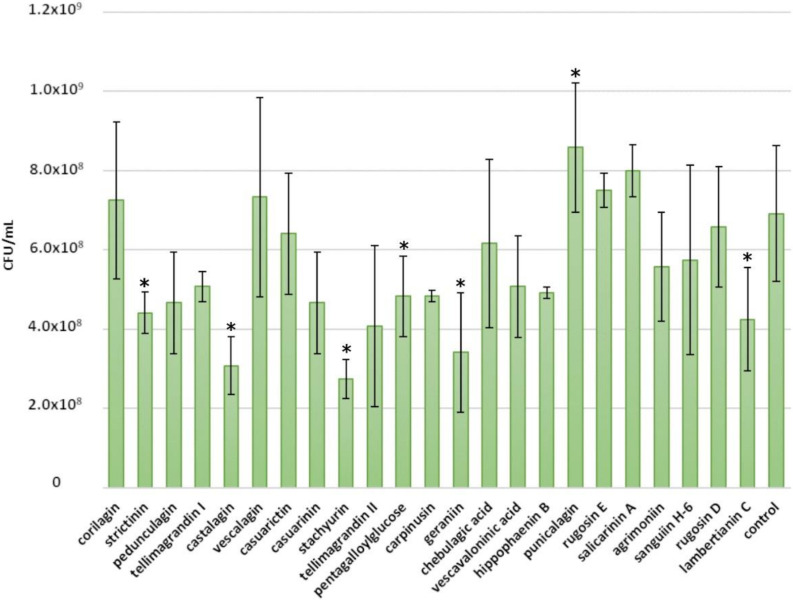
Growth of *Lactobacillus plantarum* after a 24 h treatment with 0.5 mM ellagitannin and pentagalloylglucose solutions. Growth is expressed as CFU/mL (mean values and standard deviations, *n* = 3); * *p* < 0.05.

No or very weak growth inhibition was detected for *L. plantarum*, as a growth corresponding to 10^8^ CFU/mL was observed both in the positive control and after the addition of a 0.5 mM tannin solution (Figure 8). However, even though the growth in the control seemed always to be higher, based on a paired t-test, strictinin, castalagin, stachyurin, pentagalloylglucose, geraniin, punicalagin and lambertianin C showed a minor inhibition against *L. plantarum*. In this case, the lack of growth inhibition is considered an advantage, as *Lactobacillus* comprises many probiotic species. There are several supporting previous studies conducted with different plant, fruit and berry extracts that reported either no or low growth inhibition of *Lactobacillus* by these extracts [22,23] or that observed growth inhibition only with high extract concentrations [24]. Pomegranate extract and juice containing punicalagin have been shown to even promote the growth of *Lactobacillus* [25]. However, also contradictory results have been reported, as Choe et al. (2020) showed that a sanguiin H-6-rich blackberry seed flour extract inhibited the growth of *Lactobacillus* [26].

### 2.5. Structure–Activity Relationships

Out of the four tested bacteria, *S. aureus* was found to be the most sensitive species to tannins. This observation is supported by previous studies. For example, Puupponen-Pimiä et al. (2005) have shown that ellagitannin-rich berries and berry extracts were able to inhibit the growth of *S. aureus* [8]. They have also shown in an earlier study that phenolic berry extracts could inhibit the growth of *E. coli* but not that of *Lactobacillus* [24].

Taguri et al. (1999) reported that the pyrogallol group is an important structural feature for the antimicrobial activity of polyphenols but they could not see a clear relationship between the activity and the number of pyrogallol groups [19]. Instead of considering the pyrogallol group, we focused on the number of free galloyl groups. With *S. aureus*, we could not see a clear correlation between the number of free galloyl groups and antibacterial activity, although tannins with glucopyranose cores having four or five free galloyl groups (rugosins E and D and pentagalloylglucose) inhibited the growth of *S. aureus* more extensively than the other cyclic ETs with less free galloyl groups. However, the inhibitory effects of the ET against *E. coli* and *C. perfringens* seemed to be enhanced by the additional free galloyl group.

Between-study comparisons of tannin structure and antibacterial activity of tannins are difficult, as the methods and bacterial strains vary in different studies. Additionally, several different mechanisms of action have been proposed for the antibacterial activity of tannins, for example, inhibition of extracellular microbial enzymes and oxidative phosphorylation [27] and disruption of cellular membrane permeability [21,28]. The different methods of evaluation of antibacterial potency pose its own challenges. The potency is usually evaluated by MIC values that are generally reported as µg/mL. This, however, should be taken into consideration when monomeric and oligomeric ETs are compared, as their molecular masses are different. For example, Funatogava et al. (2004) reported that monomeric ETs showed stronger antimicrobial activity against *H. pylori* than ET oligomers, most of the ET monomers having a MIC value of 12.5 µg/mL and oligomers of 25 µg/mL [20]. However, depending on the exact molecular mass of ETs, their molar potency could actually be equal. According to our results, the molecular size of ET can enhance the antibacterial activity, as oligomeric ETs were more effective in inhibiting the growth of *E. coli* and *C. perfringens* than monomeric ETs. Additionally, our results with *S. aureus* showed that the oligomeric linkage might have an effect on the antimicrobial activity, as the ET dimers with an *m*-DOG linkage exhibited stronger inhibition than ET oligomers with *m*-GOD or *m*-GOG linkages. Furthermore, we found that the poor solubility of some tannins and their ability to bind proteins make it challenging or impossible to determine their MIC values in culture solution.

It has been stated that the antibacterial potencies of tannins are less pronounced than anticipated [17,18]. However, in previous studies, corilagin was used as a model for ETs [18]. Our results show that corilagin is one of the weakest inhibitors among ETs. Other ETs are much more efficient; therefore, we can conclude that the antimicrobial effects of ETs are more significant than thought. ETs appear to be potent antimicrobials.

## 3. Materials and Methods

### 3.1. Tannin Selection, Isolation and Characterization

The extraction and isolation of ETs and pentagalloylglucose (Figure 1 and Figure 2) followed mainly our previous methods consisting in extraction, column chromatography on Sephadex LH-20 and preparative and semipreparative HPLC [11,13,29,30,31,32,33]. Twenty-two structurally different ETs were purified from different plant species. ETs were selected so that they represented different branches of the ET biosynthetic pathway, and the hydrophobicities of ETs were also taken into account [34]. Acyclic monomeric ETs: castalagin and vescalagin were isolated from purple loosestrife flowers and leaves, and vescavaloninic acid from English oak acorns [35,36,37,38,39]; stachyurin, casuarinin and hippophaenin B (α anomer) from sea buckthorn leaves [31,37,40,41]. According to Matsuo et al. (2015) [42], the stereochemistry of the NHTP group of castalagin and vescalagin was corrected to be in (*S*,*R*) configuration. Monomeric HHDP esters with a glucopyranose core, tellimagrandin I and tellimagrandin II, were isolated from meadowsweet inflorescence [30,37,43], pedunculagin from raspberry leaves [30,31,40], corilagin from *Terminalia chebula* fruits [37] and casuarictin and strictinin from sea buckthorn leaves [31,37,40]. The monomeric DHHDP esters geraniin and carpinusin were from wood cranesbill leaves and flowers [44], and modified DHHDP ester chebulagic acid from *T. chebula* fruits [37]. Punicalagin, containing the gallagyl group in its structure, was isolated from *T. chebula* fruits [37]. Pentagalloylglucose was used as a model for a hydrophobic compound and purified from tannic acid purchased from J.T. Baker (Denventer, Holland) as described in Salminen et al. (2001) [30]. The *m*-GOD oligomers, namely, dimeric sanguiin H-6 and trimeric lambertianin C, were isolated from raspberry leaves [31,45]; the *m*-DOG dimers, rugosins D and E, were from meadowsweet inflorescence [46], the *m*-GOG dimer agrimoniin was from silverweed leaves [31,37,47], and the acyclic dimer, salicarinin A, from purple loosestrife flowers and leaves [30,40,48]. The structures were confirmed by UPLC–DAD–ESI-MS and NMR spectroscopy based on Karonen et al. [13,33]. ^1^H-NMR spectra of the ETs and pentagalloylglucose are presented in Supplementary Materials (Figures S1–S23).

### 3.2. Bacterial Culture Conditions

Four different organisms were used for the antimicrobial tests: *E. coli* (APEC46), *C. perfringens* (NCTC 8678 from human faeces) and *L. plantarum* (NCIMB 30187 from pickled cabbage); a methicillin-susceptible strain of *S. aureus* was isolated from ham (National Agricultural Research Foundation, Lycovrissi, Greece) [49]. MRS broth and plates were used for the cultivation of *L. plantarum*, and Luria–Bertani (LB) broth and plates were used for the cultivation of *E. coli*, *S. aureus* and *C. perfringens*. *E. coli* and *S. aureus* were cultivated aerobically at 37 °C, whereas *C. perfringens* and *L. plantarum* were cultivated anaerobically at 37 °C. Stock cultures were maintained at −80 °C. Before antimicrobial testing, the cultures were transferred from the stock culture on solid media and incubated for one day.

### 3.3. Antimicrobial Testing of ETs

An inoculum of bacterial culture was grown overnight on LB/MRS plates. A dilution of bacteria was made from the plate in LB/MRS broth, and the optical density of the dilution at 600 nm was adjusted to 0.010. The inhibitory assay was performed on a flat-bottomed 96-well plate. Then, 50 µL of LB/MRS broth, 100 µL of tannin solution (1 mM in LB/MRS broth with 5% ethanol) and 50 µL of the bacteria solution were added to each well. The total volume was 200 µL, and the final tannin concentration was 0.5 mM. In addition, 100 µL of LB/MRS broth with 5 % ethanol was added to the positive control instead of the tannin solution. Three replicates were used in the experiment. The tannin concentration of 0.5 mM was chosen to avoid tannin-induced precipitation of broth proteins.

The plate was incubated at 37 °C for 24 h without shaking. After 24 h, a 50 µL sample was taken, diluted in LB/MRS broth and plated on LB/MRS plates. The plates were incubated overnight at 37 °C, and colonies were counted approximately after 24 h, except for *S. aureus*, for which the colonies were counted after 48 h.

### 3.4. Statistical Analysis

A paired t-test was used for statistical analysis, and a *p* value <0.05 was considered statistically significant. To estimate if there is a correlation between the molecular size of ETs and their antimicrobial strength, the bacterial growth after ET treatment (CFU/mL) was plotted against the molecular weight of the ET and fitted with a linear regression model.

## 4. Conclusions

All ETs were shown to exhibit antimicrobial effects. The strength for the growth inhibition was in the following order: *S. aureus* > *E. coli* > *C. perfringens*. The structural features of ellagitannins that affected most clearly their antimicrobial activity were the molecular size for *E. coli* and *C. perfringens*, the molecular flexibility for *C. perfringens* and the type of oligomeric linkage for *S. aureus*. ETs did not exert unwanted antimicrobial effects against probiotics, as no or very weak inhibition was observed for *L. plantarum*.

## Supplementary Materials

^1^H-NMR spectra of the ellagitannins and pentagalloylglucose tested for their antimicrobial activity (Figures S1–S23).
