# Supplementary material for: Antimicrobial Activities of Ellagitannins against Clostridiales perfringens, Escherichia coli, Lactobacillus plantarum and Staphylococcus aureus
Source: Molecules. 2020 Aug 14;25(16):3714. doi: 10.3390/molecules25163714 (PMC7465317; doi:10.3390/molecules25163714)
Supplement: Supplementary file 1 [file molecules-25-03714-s001.pdf]

molecules

Supplementary Material

Antimicrobial Activities of Ellagitannins against *Clostridiales perfringens*, *Escherichia coli*, *Lactobacillus plantarum* and *Staphylococcus aureus*

Elina Puljula [1], Gemma Walton [2], Martin J. Woodward [2,3] and Maarit Karonen [1,*]

[1] Natural Chemistry Research Group, Department of Chemistry, University of Turku, FI-20014 Turku, Finland; elina.puljula@gmail.com
[2] Department of Food and Nutritional Studies, The University of Reading, Reading RG6 6AH, UK; g.e.walton@reading.ac.uk (G.W.); m.j.woodward@reading.ac.uk or mjw@foliumscience.com (M.J.W.)
[3] Folium Science, Unit-DX, Bristol BS2 OXJ, UK
* Correspondence: maarit.karonen@utu.fi; Tel.: +358-29-450-3179

Contents

Figure S1. ^{1}H-NMR spectrum of corilagin ... 2
Figure S2. ^{1}H -NMR spectrum of strictinin ... 2
Figure S3. ^{1}H -NMR spectrum of pedunculagin ... 3
Figure S4. ^{1}H -NMR spectrum of tellimagrandin I ... 3
Figure S5. ^{1}H -NMR spectrum of castalagin ... 4
Figure S6. ^{1}H -NMR spectrum of vescalagin ... 4
Figure S7. ^{1}H -NMR spectrum of casuarictin ... 5
Figure S8. ^{1}H -NMR spectrum of casuarinin ... 5
Figure S9. ^{1}H -NMR spectrum of stachyurin ... 6
Figure S10. ^{1}H -NMR spectrum of tellimagrandin II ... 6
Figure S11. ^{1}H -NMR spectrum of pentagalloylglucose ... 7
Figure S12. ^{1}H -NMR spectrum of carpinusin ... 7
Figure S13. ^{1}H -NMR spectrum of geraniin ... 8
Figure S14. ^{1}H -NMR spectrum of chebulagic acid ... 8
Figure S15. ^{1}H -NMR spectrum of vescavaloninic acid ... 9
Figure S16. ^{1}H -NMR spectrum of hippophaenin B ... 9
Figure S17. ^{1}H -NMR spectrum of punicalagin ... 10
Figure S18. ^{1}H -NMR spectrum of rugosin E ... 10
Figure S19. ^{1}H -NMR spectrum of salicarinin A ... 11
Figure S20. ^{1}H -NMR spectrum of agrimoniin ... 11
Figure S21. ^{1}H -NMR spectrum of sanguiin H-6 ... 12
Figure S22. ^{1}H -NMR spectrum of rugosin D ... 12
Figure S23. ^{1}H -NMR spectrum of lambertianin C ... 13

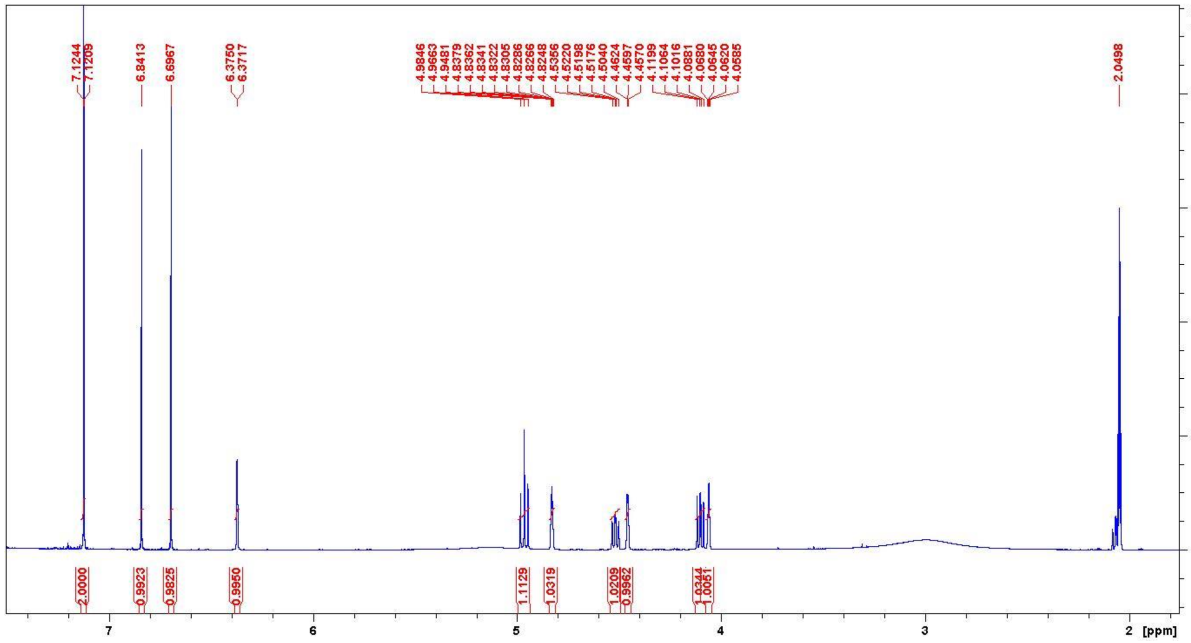

Figure S1. ^{1}H-NMR spectrum of corilagin

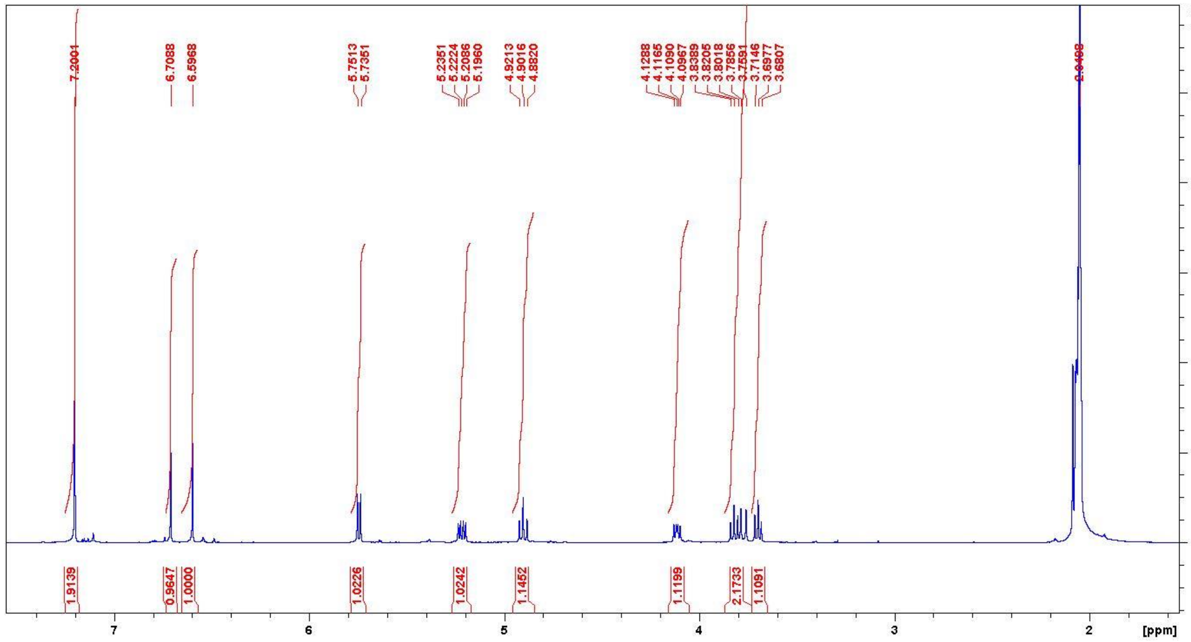

Figure S2. ^{1}H -NMR spectrum of strictinin

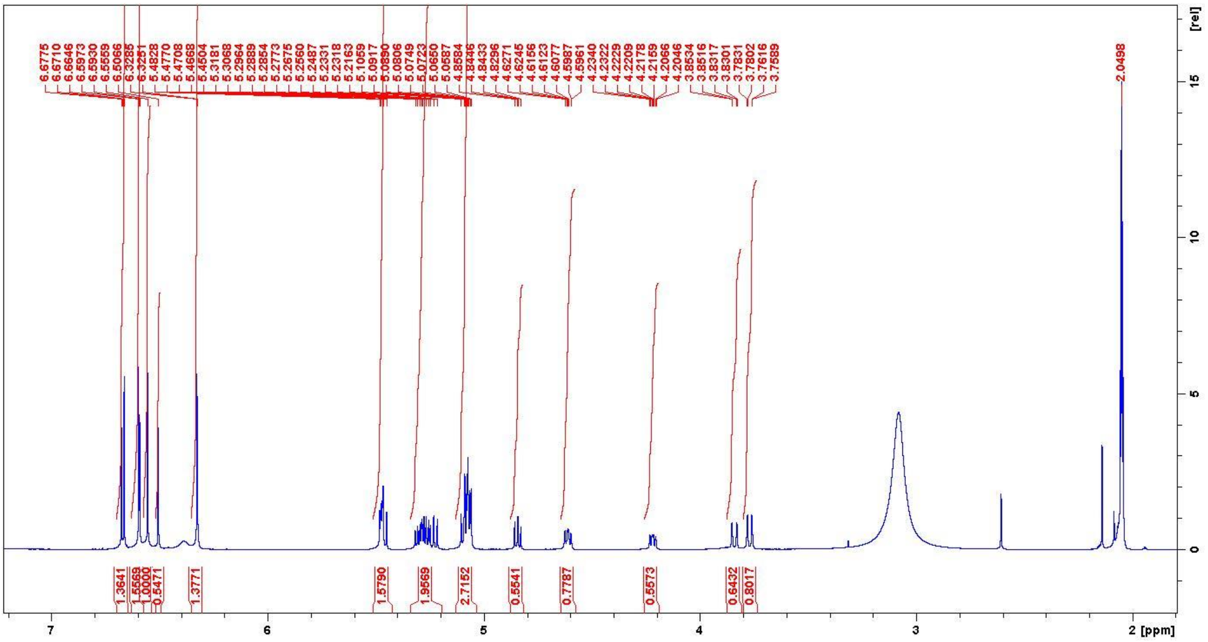

Figure S3. ^{1}H -NMR spectrum of pedunculagin

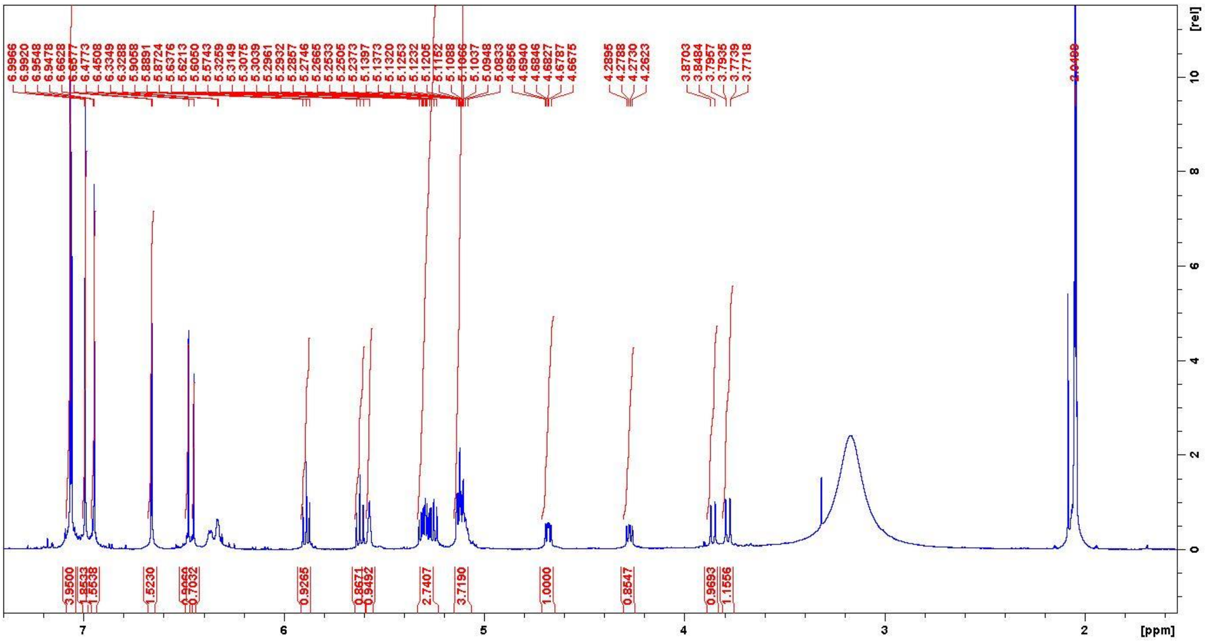

Figure S4. ^{1}H -NMR spectrum of tellimagrandin I

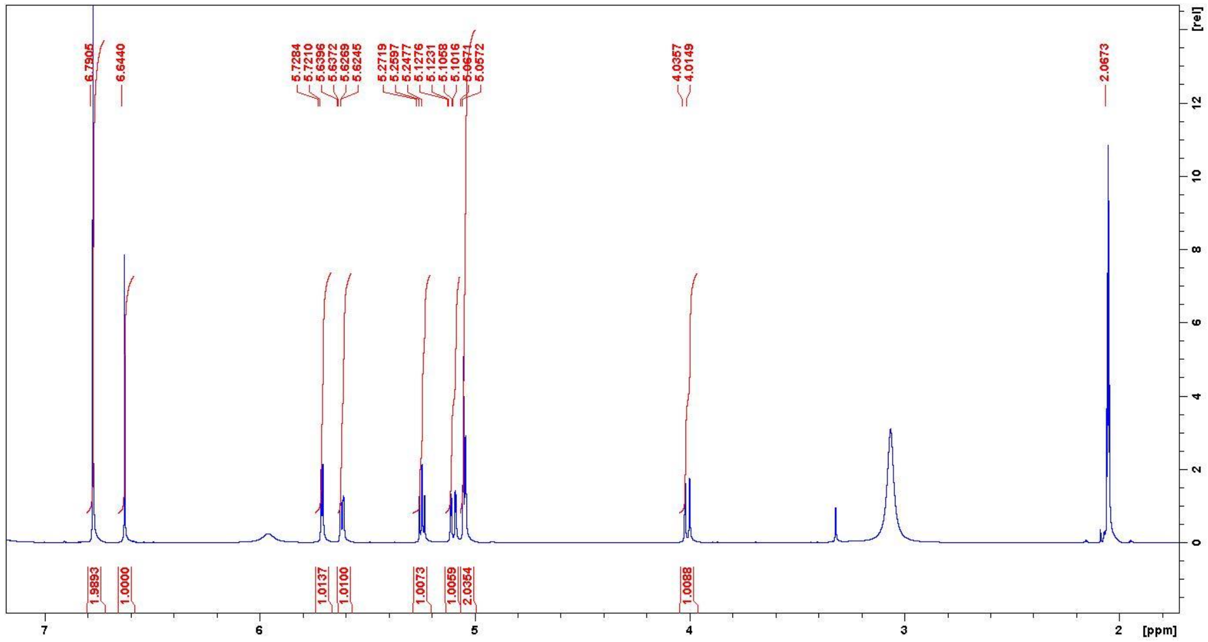

Figure S5. ^{1}H -NMR spectrum of castalagin

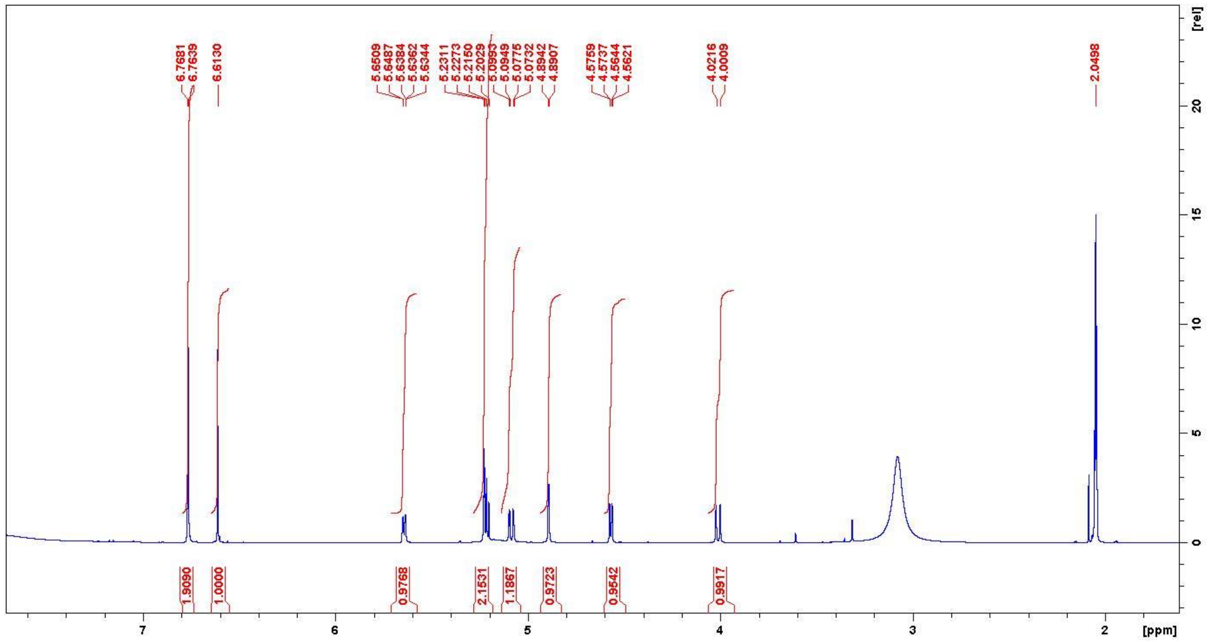

Figure S6. ^{1}H -NMR spectrum of vescalagin

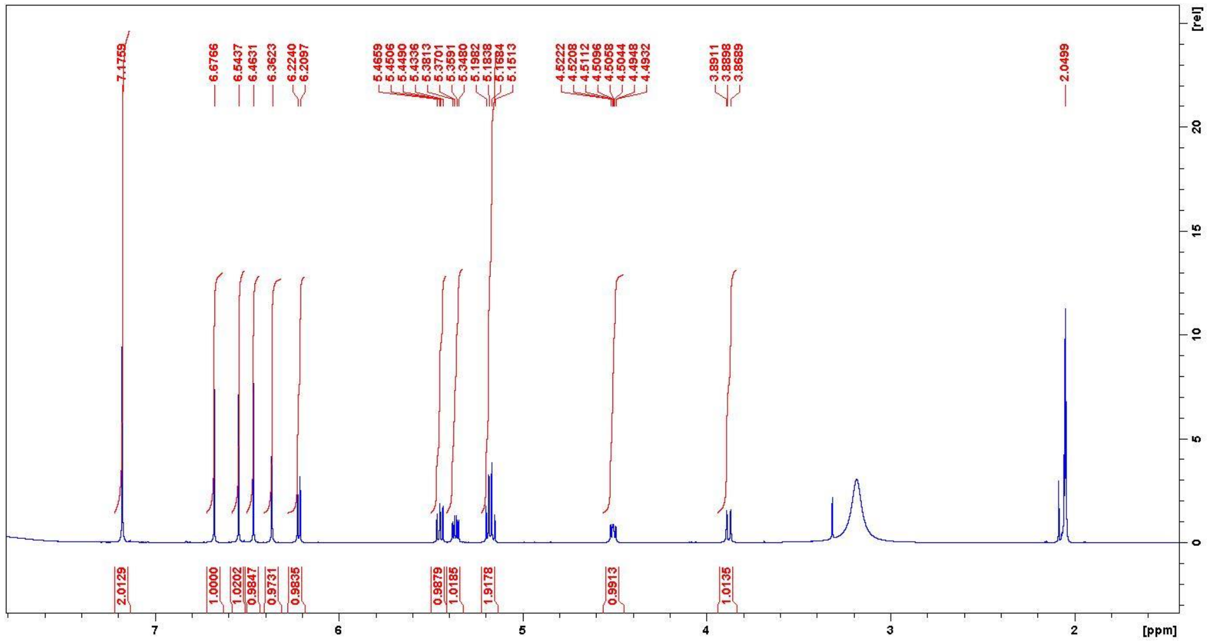

Figure S7. ^{1}H -NMR spectrum of casuarictin

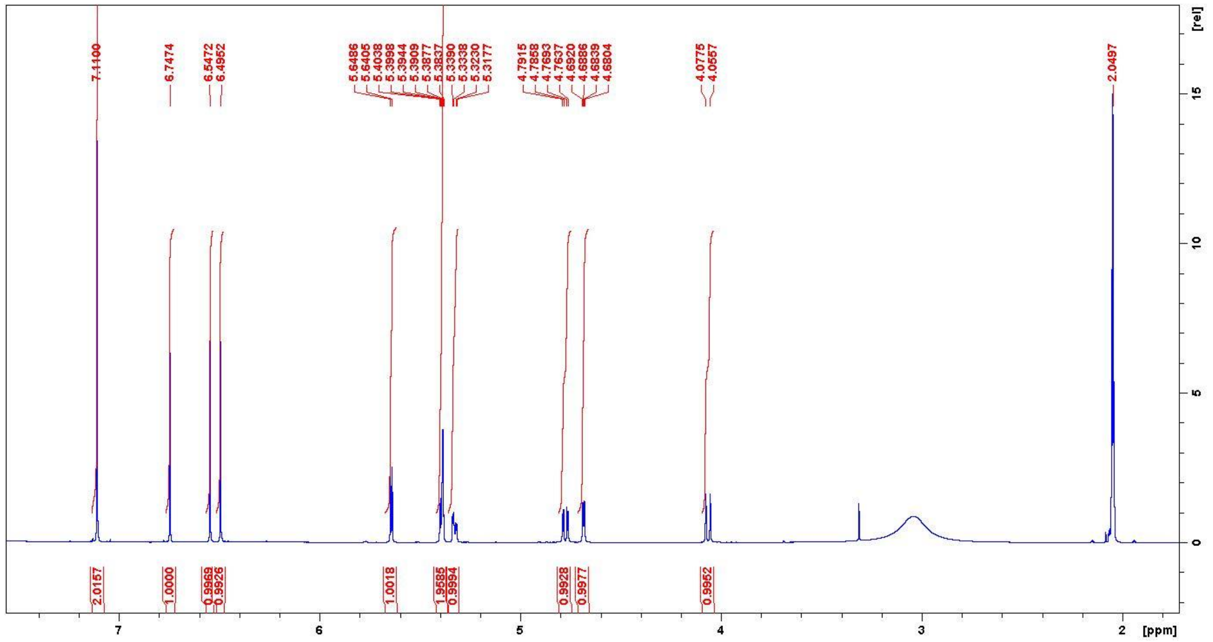

Figure S8. ^{1}H -NMR spectrum of casuarinin

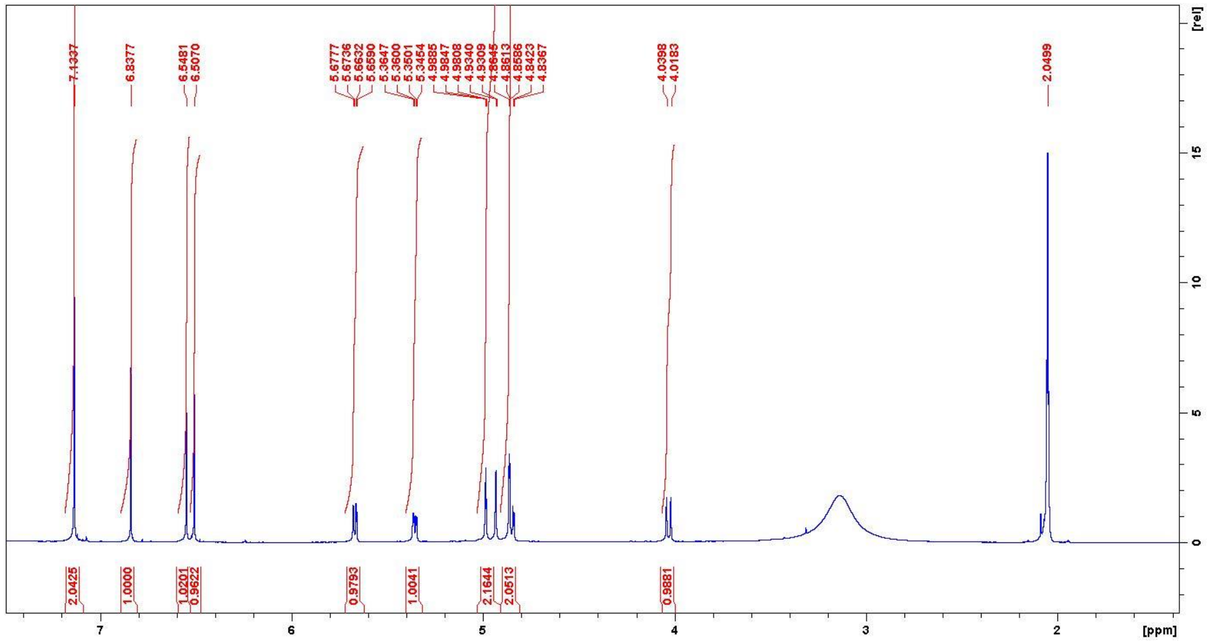

Figure S9. ^{1}H -NMR spectrum of stachyurin

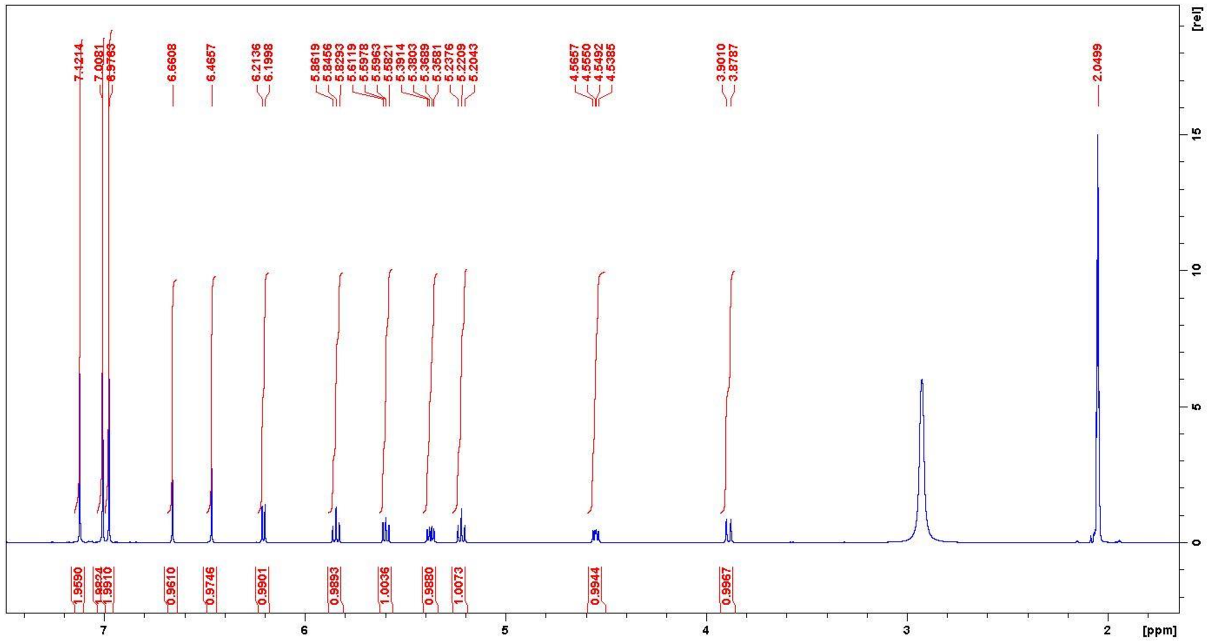

Figure S10. ^{1}H -NMR spectrum of tellimagrandin II

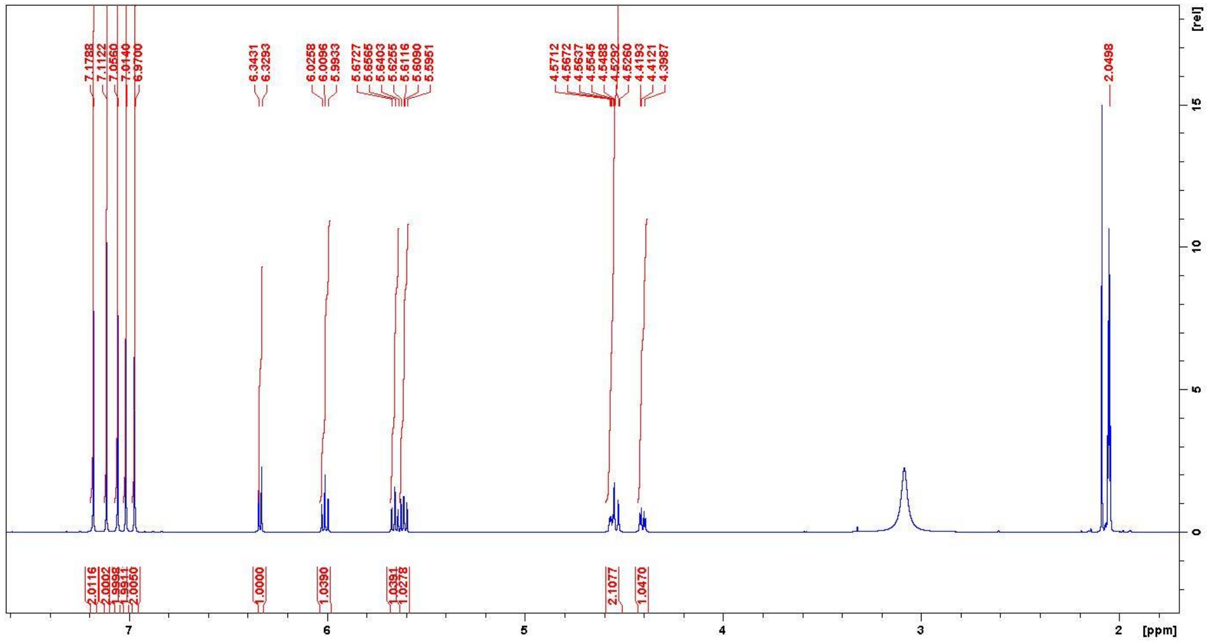

Figure S11. ^{1}H -NMR spectrum of pentagalloylglucose

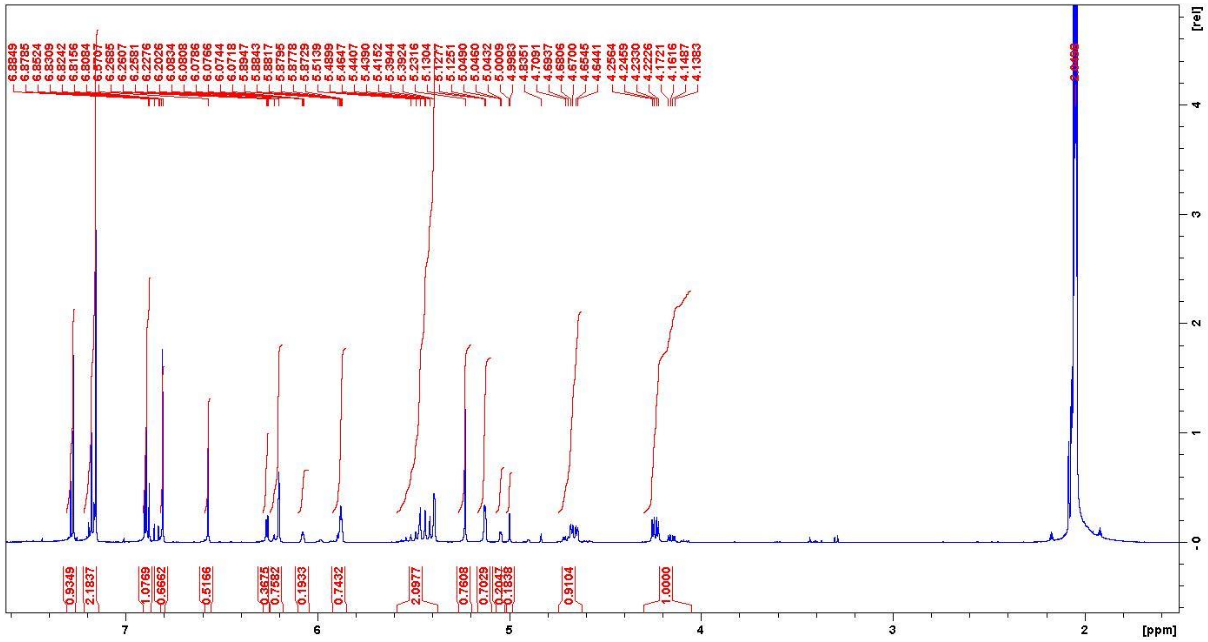

Figure S12. ^{1}H -NMR spectrum of carpinusin

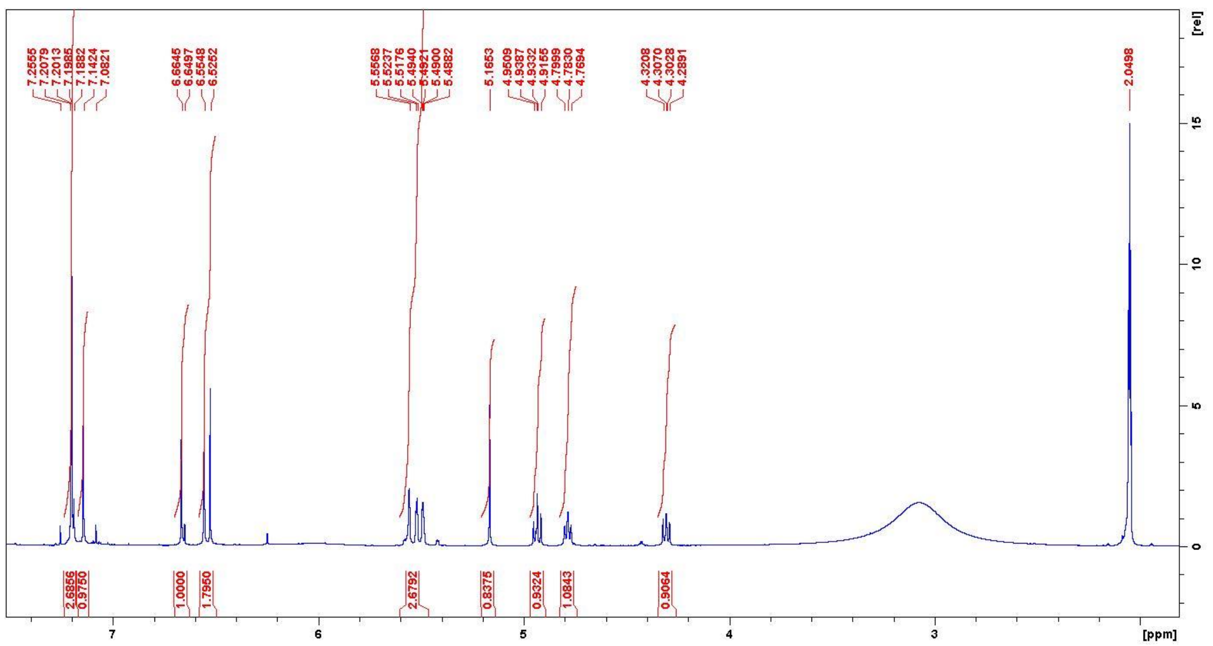

Figure S13. ^{1}H -NMR spectrum of geraniin

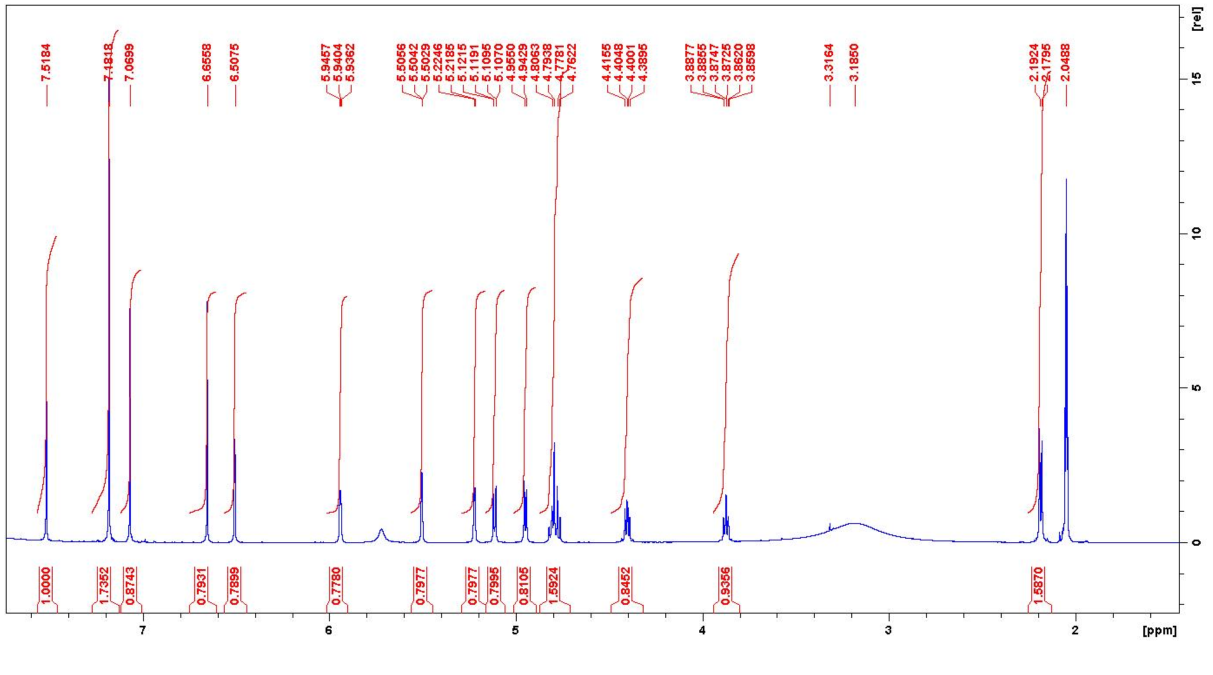

Figure S14. ^{1}H -NMR spectrum of chebulagic acid

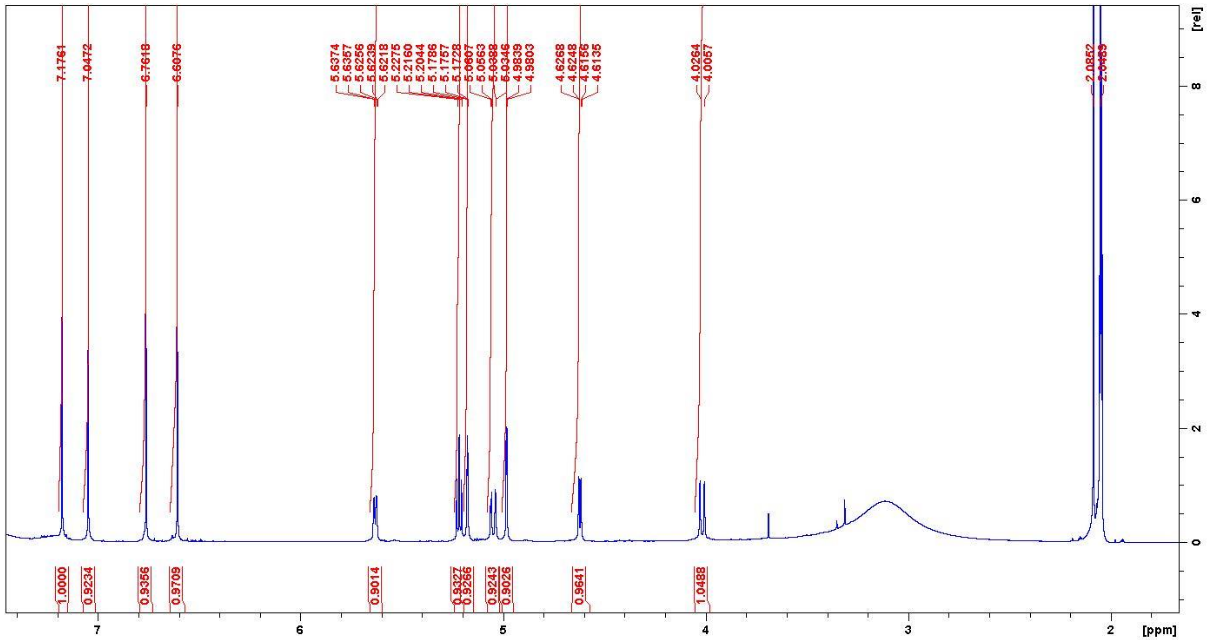

Figure S15. ^{1}H -NMR spectrum of vescavaloninic acid

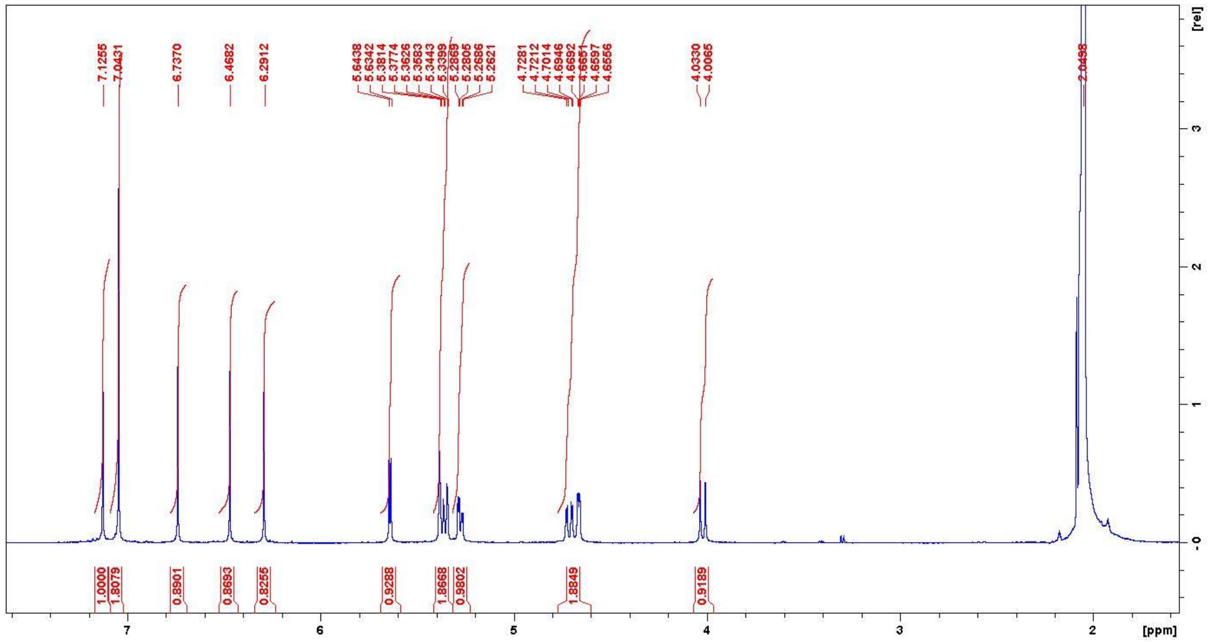

Figure S16. ^{1}H -NMR spectrum of hippophaenin B

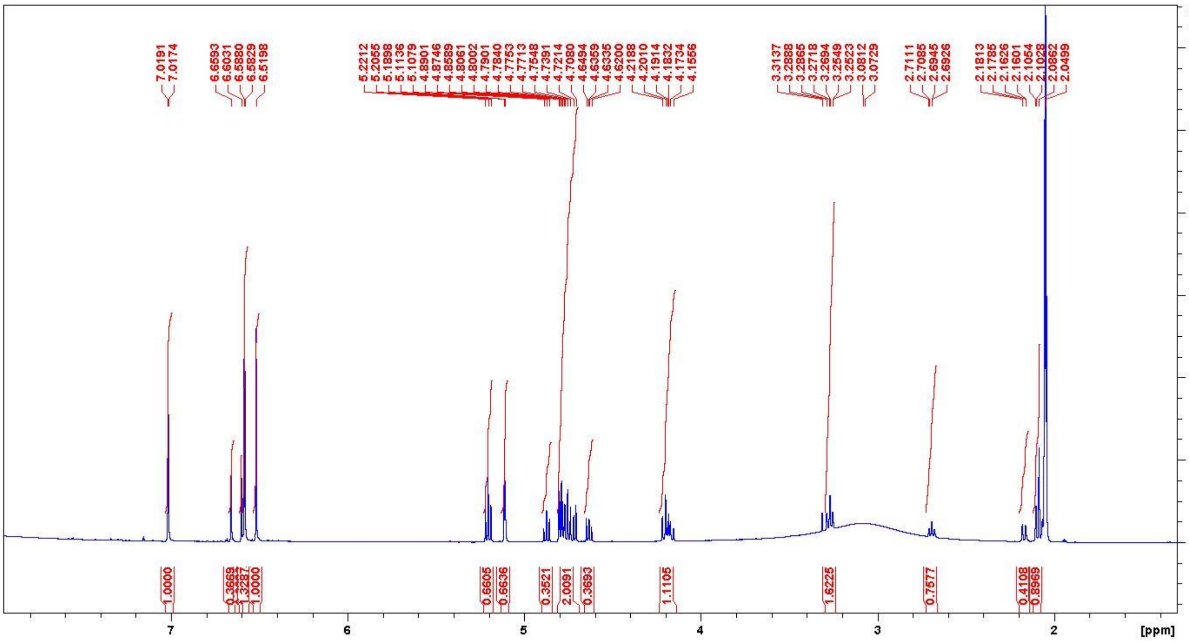

Figure S17. ^{1}H -NMR spectrum of punicalagin

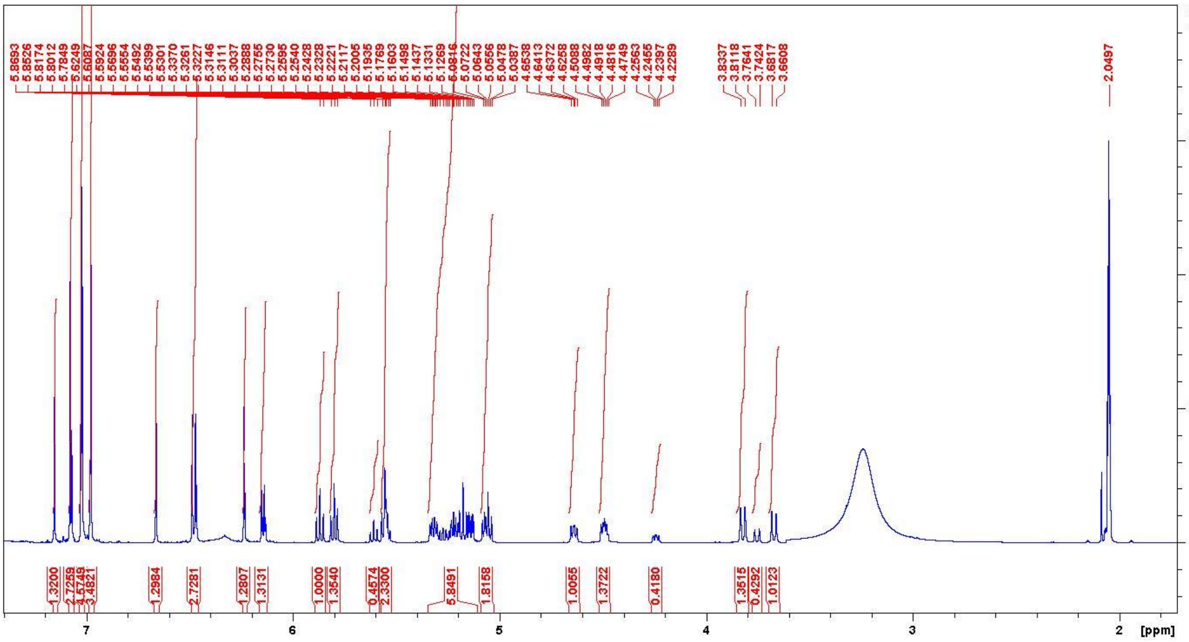

Figure S18. ^{1}H -NMR spectrum of rugosin E

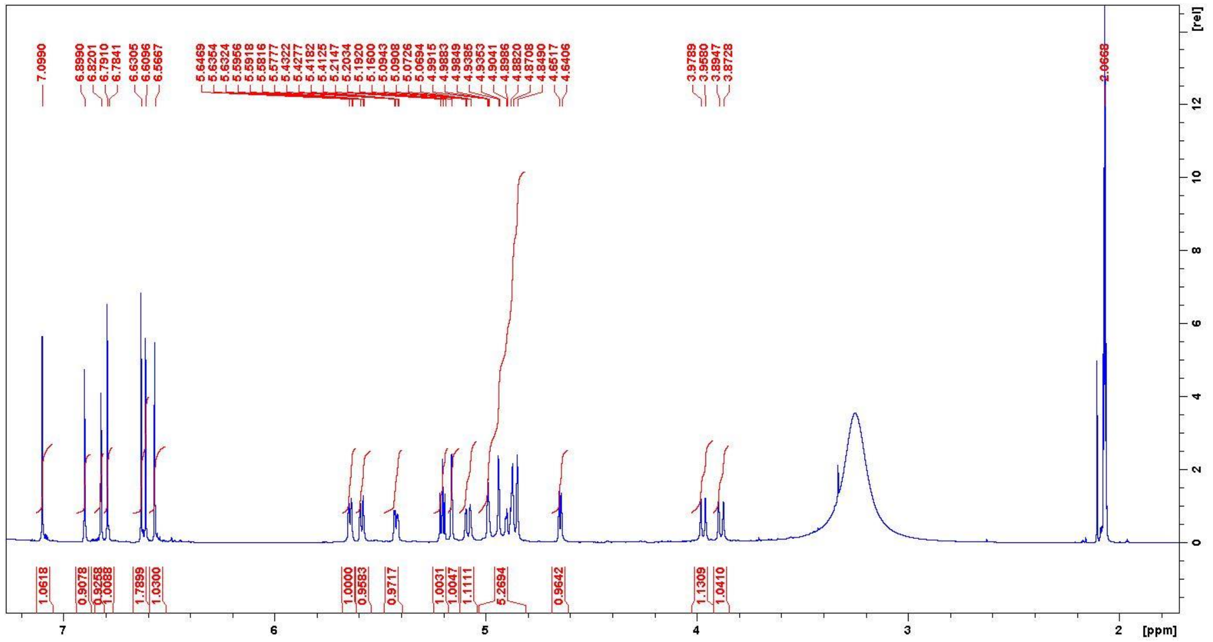

Figure S19. ^{1}H -NMR spectrum of salicarinin A

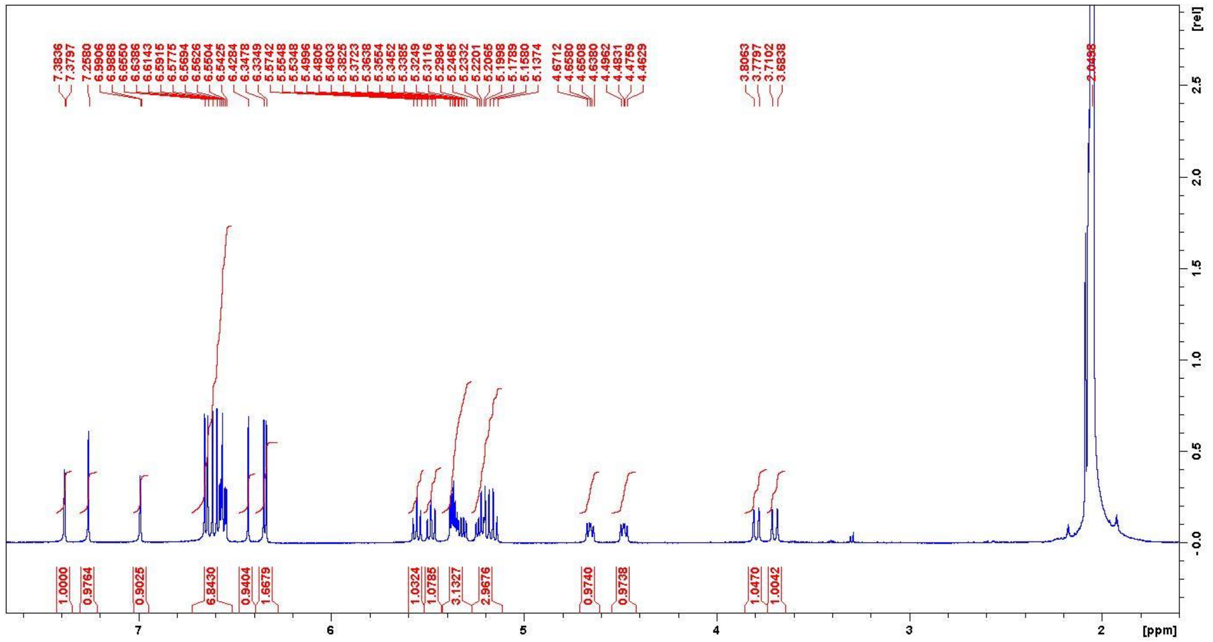

Figure S20. ^{1}H -NMR spectrum of agrimoniin

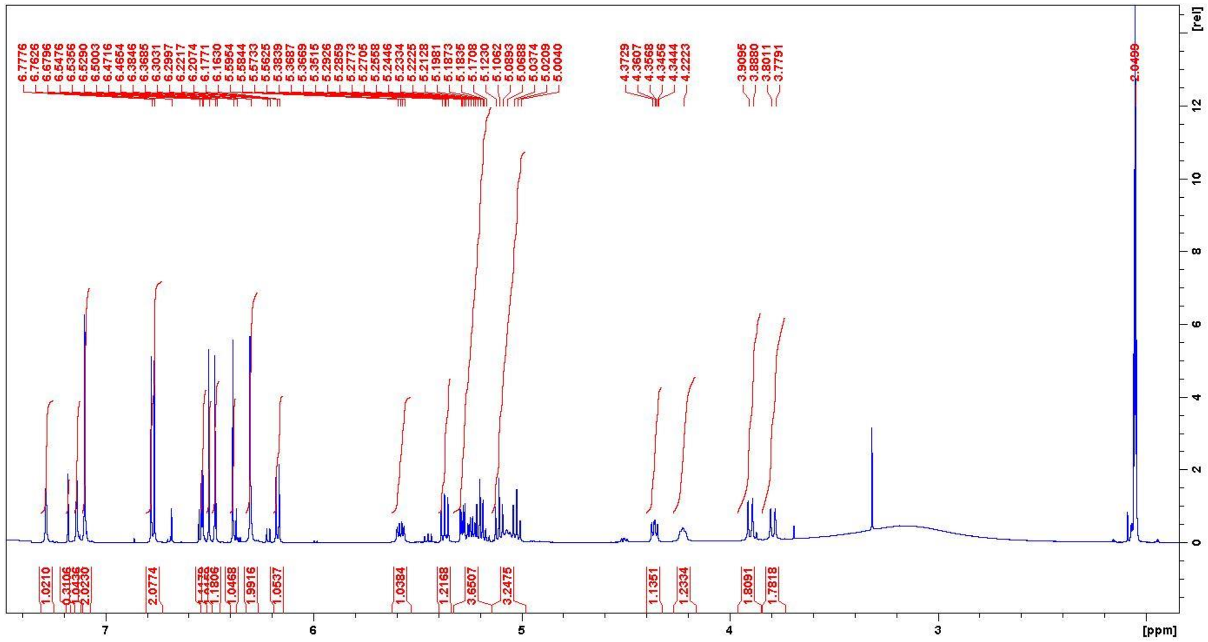

Figure S21. ^{1}H -NMR spectrum of sanguiin H-6

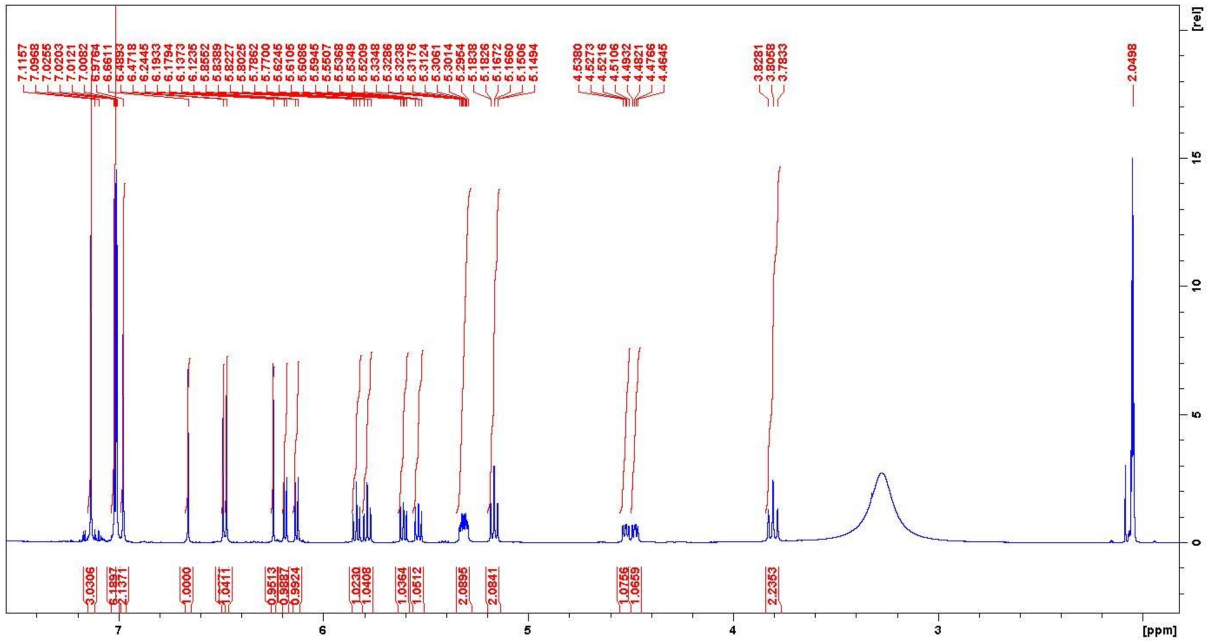

Figure S22. ^{1}H -NMR spectrum of rugosin D

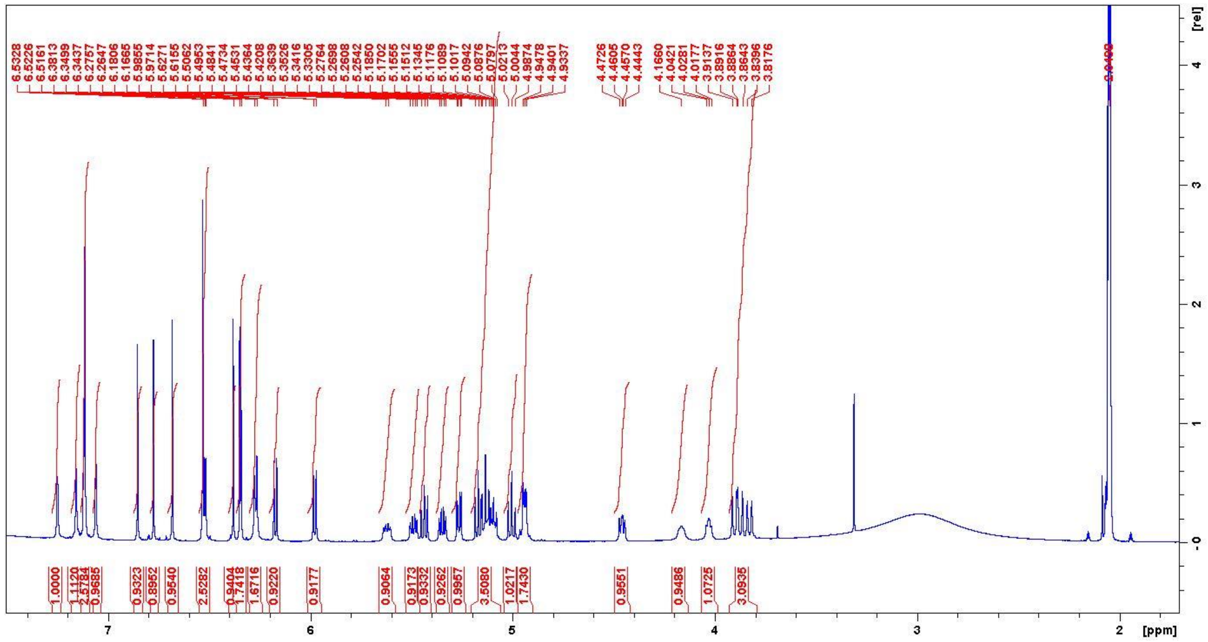

Figure S23. ^{1}H -NMR spectrum of lambertianin C
